# Optimized intelligent control of a 2-degree of freedom robot for rehabilitation of lower limbs using neural network and genetic algorithm

**DOI:** 10.1186/1743-0003-10-96

**Published:** 2013-08-14

**Authors:** Wahab Aminiazar, Farid Najafi, Mohammad Ali Nekoui

**Affiliations:** 1 Department of Electrical Engineering, Science and Research branch, Islamic Azad University, Tehran, Iran; 2 Department of Mechanical Engineering, Engineering Faculty, Guilan University, Rasht, Iran; 3 Department of Electrical Engineering, K.N.Toosi. University of Technology, Tehran, Iran

**Keywords:** Rehabilitation, Robotic rehabilitation, Intelligent control, Impedance control, Adaptive control, Neural network, Genetic algorithm

## Abstract

**Background:**

There is an increasing trend in using robots for medical purposes. One specific area is rehabilitation. Rehabilitation is one of the non-drug treatments in community health which means the restoration of the abilities to maximize independence. It is a prolonged work and costly labor. On the other hand, by using the flexible and efficient robots in rehabilitation area, this process will be more useful for handicapped patients.

**Methods:**

In this study, a rule-based intelligent control methodology is proposed to mimic the behavior of a healthy limb in a satisfactory way by a 2-DOF planar robot. Inverse kinematic of the planar robot will be solved by neural networks and control parameters will be optimized by genetic algorithm, as rehabilitation progress.

**Results:**

The results of simulations are presented by defining a physiotherapy simple mode on desired trajectory. MATLAB/Simulink is used for simulations. The system is capable of learning the action of the physiotherapist for each patient and imitating this behaviour in the absence of a physiotherapist that can be called robotherapy.

**Conclusions:**

In this study, a therapeutic exercise planar 2-DOF robot is designed and controlled for lower-limb rehabilitation. The robot manipulator is controlled by combination of hybrid and adaptive controls. Some safety factors and stability constraints are defined and obtained. The robot is stopped when the safety factors are not satisfied. Kinematics of robot is estimated by an MLP neural network and proper control parameters are achieved using GA optimization.

## Background

The process of strengthening muscles to their normal values is a costly labor which requires time and patience [1]. This process is named rehabilitation. An intelligent instrument that replaces the duty of the physiotherapist and can accomplish such routine physical movements without the guidance and assistance of a physiotherapist will simplify the process and lower the costs drastically [2]. There are many exercise machines for rehabilitation purposes like CPMs [2]. Nevertheless, these machines are used only for ankle function and because of their low degree of freedom, their poor dynamic efficiency and prospect of high expense, they are used limitedly [3,4]. The most important machines used widely in many medical centers for therapy and rehabilitation purposes are LOKOMAT [5], ALEX [6] and LOPES [7]. These machines have high degree of freedom but their high cost causes them to be used limitedly. Moreover, their manipulation is hard and requires ingenuity. In addition, control system design is one of the major difficulties in construction of rehabilitation robots. Different approaches were developed to control movement of robot-aided therapy attached to human limbs [1,8-12]. It is observed that the devices developed for rehabilitation purpose usually employ two control methods including hybrid control (force and position control) and impedance control. Intelligent techniques, which are optimized based on therapy session, were used in few works [2,9]. The main purpose of the developed system in this study is to introduce a low-cost system to satisfy the patient safety by a flexible structure controlled by an intelligent control strategy. The control parameters will be changed based on the therapy of different stages and patient qualification, thus the hybrid and adaptive control are used for controlling the suggested system. Neural networks are employed as the reference input of the proposed controller. Control parameters are optimized based on therapy sessions and safety factors and for this purpose a genetic evolutionary algorithm was applied. The suggested system can be used for rehabilitation of two limbs/joints (knee and hip).

## Methods

### Rehabilitation mode

**Figure 1:**
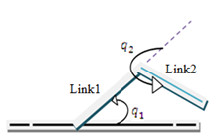
**A planar 2-DOF robot for proposed rehabilitation mode based on flexion and extension movement.** Link 1 is aligned to the thigh and link 2 is aligned to the shank and q_1٫_q_2_ are the angles of hip and knee, respectively.

The proffered structure is based on the flexion and extension movement for knee and hip [1]. For this purpose a 2-DOF planar robot is defined that can be attached to the trunk of lower limb (Figure 1).

In Figure 1, link 1 is aligned to the thigh and link 2 is aligned to the shank and *q*_1_٫*q*_2_ are the angles of hip and knee, respectively and the limits of them are based on the flexion-extension of knee and hip process shown in Figure 1 and are written as:

(1) $$0\leq q_{1}\leq \frac{\pi }{2}$$

(2) $$\frac{7\pi }{6}\leq q_{2}\leq 2\pi$$

In robotic rehabilitation, the desired trajectory of manipulator obtained from the physiotherapist and then the related variables of the robot such as angles and velocity of them are computed based on inverse kinematic problem. Thereafter these parameters are used for control of robot to track the desired trajectory. These issues will be described in the next stages.

### Control strategy used in proposed algorithm

As mentioned earlier, neural networks are employed as the reference input of proposed control algorithm. Thus, the neural network and its usages in proposed strategies are explained first and then the proffered control strategy will be described.

### Neural network

An important area of neural networks application is in the field of robotics. Usually, these networks are designed for learning and reconstructing complex non-linear mapping and have been widely used in the identification and control of a manipulator, which is the most important form of an assistant robot, and in tracking a trajectory based on sensory information. Generally, kinematics of parallel robot are non-linear problems and difficult to solve, thus an MLP neural network is used to estimate the joint variables. The second idea in using neural networks is originated from the results of experiments showing that there are training vulnerability centers in the adult mammalian spinal cord which activate and control motor neurons that are responsible for walking patterns [13-15]. These walking patterns that have been previously been reserved in can be replaced by other neurons. It means each neuron can be considered as a walking pattern.

The MLP neural network used in suggested method has two layers *tansig* activation function in layer (1) and activation *purelin* function in layer (2). The best number of neurons in layer (1) is obtained from an iteration algorithm. The Levenberg-Marquardt back propagation or *trainlm* algorithm is used for network training.

### Control strategy

Control strategies of rehabilitation systems can be classified into three categories: force control, position control, position and force control [16,17]. Nevertheless, unlike industrial robots, rehabilitation-aided robots must be configured for stable, safe and compliant motion while interacting with humans [18]. The impedance control strategy propounded by Hogan [17,19] is one of the most appropriate approaches for such applications. Impedance control aims at controlling the position and force by adjusting the mechanical impedance of the manipulator to the external forces generated by contact with the manipulator’s environment. Mechanical impedance is roughly an extended concept of the stiffness of a mechanism against a force applied to it [20].

**Figure 2:**
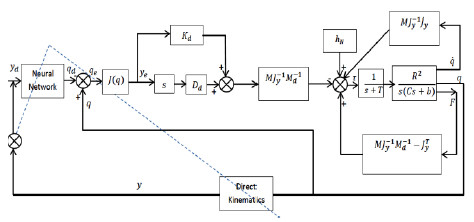
The impedance control block diagram.

The control block diagram used for rehabilitation purpose shown in Figure 2.

Therefore, the necessary joint torques to obtain desired impedance parameters are computed as: (Eq. (3) is obtained from the dynamic equation of robot manipulator that is in contact with its environments in joint space [2]):

(3) $$\begin{array}{l} \tau =h_{N}\left(q,\dot{q}\right)-M\left(q\right)J_{y}^{-1}\left(q\right){\dot{J}}_{y}\left(q\right)\dot{q}-M\left(q\right)J_{y}^{-1}\left(q\right)M_{d}^{-1}\left(D_{d}{\dot{y}}_{e}+K_{d}y_{e}\right)\\ \;+\left[M\left(q\right)J_{y}^{-1}\left(q\right)M_{d}^{-1}-J_{y}^{T}\right]F\; \end{array}$$

Where the *y* and subscript *y* denote the task space and the *q* denotes the joint space.

In this equation τ^2*1^ is the torque input vector, *q*_*d*_^2*1^ is the joint vector, *y*^2*1^ is the manipulator’s end effector vector, *q*^2*1^ is the joint angle vector, *h*_*N*_(*q*٫˙*q*)^2*1^ is the Coriolis and centrifugal force effects and other effects (such as gravity), *M*(*q*)^2*2^ is the inertia matrix, *M*_*d*_^2*2^ is the desired inertia matrix, *J*^2*2^ is the Jacobean matrix, *D*_*d*_^2*2^ is the desired damping coefficient matrix, *K*_*d*_^2*2^ is the desired stiffness coefficient matrix and *F*^2*1^ is external force exerted on the end-effector by its environment (this force can be defined as action and reaction force between patient and end-effector).

The term $\frac{R^{2}}{s\left(\mathrm{Cs}+b\right)}$ denotes the transfer function of any arm of robot, equipped with a DC servo motor, where R is the gear reduction ratio in motor and the parameters C and b are the effective moment of inertia and viscous friction coefficient, respectively [15]. $\frac{1}{s+T}$ is the amount of approximated delay.

In the applied control structure, Neural Network box is used to convert *y*_*d*_ (desired position) to *q*_*d*_ (desired joint angles). And *y* is the target of NN. In this block diagram, it is assumed that:

(4) $$y_{e}=\mathrm{dy}=J\left(q\right)\mathrm{dq}\;≅\;J\left(q\right)q_{e}$$

Where γ_*e*_ represents the error, or deflection of the MP (*y*) from its reference/desired position (*y*_*d*_) and *q*_*e*_ represents the error, or deflection of the joints (*q*) from its desired position (*q*_*d*_).

### Patient safety in the proposed algorithm

Patient safety is one of the most important factors in rehabilitation systems and can be guaranteed by the stability of software and hardware. Stability conditions for robotic systems under impedance or hybrid controllers had been investigated in some researches [1,8,17,19]. In this paper, a new asymptotic stability conditions for stiffness and impedance controllers is applied using an appropriate Routh approach [15] based on the relationship between a joint angle of the robot and desired trajectory. Corresponding transfer function can be defined as:

(5) $$G\left(s\right)=\frac{C\left(s\right)}{R\left(s\right)}=\frac{q}{q_{d}}$$

According to Eq. (3) and the following substitutions:

(6) $$F=Ky_{e}$$

(7) $$Ф=M\left(q\right)J_{y}^{-1}\left(q\right)$$

(8) $$h_{N}\left(q,\dot{q}\right)=\mathrm{Nq}$$

Where *g* is the gravitational acceleration, *m* is the mass of patient leg, L_*g*_ is distance between the joint and the mass center of link and N is the effects (Coriolis and centrifugal force effects and other effects such as gravity) without static (coulomb) friction (friction ignored).

The transfer function of Eq. (3) will be:

(9) $$\begin{array}{l} G\left(s\right)=\frac{C\left(s\right)}{R\left(s\right)}=\frac{q}{q_{d}}\\ \;=\frac{\left(ФM_{d}^{-1}D_{d}J\right)s+ФM_{d}^{-1}K_{d}J-ФM_{d}^{-1}\mathrm{KJ}+J^{T}\mathrm{KJ}}{s^{2}\left(\mathrm{ФJ}+\mathrm{RC}\right)+s(\mathrm{TRC}+1+ФM_{d}^{-1}D_{d}J)}\\ \;+(ФM_{d}^{-1}K_{d}J-ФM_{d}^{-1}\mathrm{KJ}+J^{T}\mathrm{KJ}+N) \end{array}$$

The denominator polynomial is:

(10) $$\begin{array}{l} d\left(s\right)=s^{2}\left(\mathrm{ФJ}+\mathrm{RC}\right)\\ \;+s(\mathrm{TRC}+1+ФM_{d}^{-1}D_{d}J)\\ \;+(ФM_{d}^{-1}K_{d}J-ФM_{d}^{-1}\mathrm{KJ}+J^{T}\mathrm{KJ}+N)\\ \;=a_{0}s^{2}+a_{1}s+a_{2} \end{array}$$

After determining the stability conditions of controller gains based on Routh’s theory [15], and taking into account that (*M,K,D*) are positive definite matrices, there will be:

(11) $$\begin{array}{l} \mathrm{RC}>-\mathrm{ФJ}\;,\;ФM_{d}^{-1}K_{d}J+J^{T}\mathrm{KJ}+N\\ \;>ФM_{d}^{-1}\; \end{array}$$

If we consider one of the joints of suggested robot (knee joint) and small movement these substitutions will be obtained:

M=I,Jy=JyT=Lg,Jy−1=1Lg

$$h_{n}=\tau_{\mathrm{gravity}}=\mathrm{mgsin}\left(q\right)L_{g}=\mathrm{mgq}L_{g}$$

Dd,Kd=I,Md=12I,Lg=1m,g=−10ms2

Now the transfer function of (5) will be:

(12) $$G\left(s\right)=\frac{-2sR^{2}+kR^{2}-2R^{2}}{s^{3}\left(-C\right)+s^{2}\left(-\mathrm{TC}-b\right)+s\left(-\mathrm{bT}-2R^{2}\right)+R^{2}\left(10m-2+k\right)}$$

The denominator polynomial is:

$$\begin{array}{l} d\left(s\right)=s^{3}\left(-C\right)+s^{2}\left(-\mathrm{TC}-b\right)+s\left(-\mathrm{bT}-2R^{2}\right)\\ \;+R^{2}\left(10m-2+k\right) \end{array}$$

Then the stability condition will be:

(13) $$k>\frac{2TR^{2}-10\mathrm{mT}R^{2}-\left(\mathrm{TC}+b\right)\left(2R^{2}+\mathrm{bT}\right)}{TR^{2}}$$

As shown in the next sections, the deviation of actual path from the desired path is considered as another system stability condition. In this paper, safety is guaranteed since some of the controller parameters can be adapted under the following criteria:

1- The stability constraints in (11) or (13)

2- Desired deviation or difference between actual and desired path (Δ*P*_*d*_ will be explained in next section).

3- Different stroked patients (obtained from physiotherapist).

4- Different states of progression in the therapy process (by progress of rehabilitation steps and improvement in movement or feeling less pain obtained from physiotherapist).

5- The action/reaction force (F) between patient and robot (by a force sensor).

The robot is stopped when the safety factors is not satisfied. Thus, the recommended control strategy will be based on the combination of two strategies: impedance control and adaptive strategy. Controller parameters are finely tuned using a constrained non-linear optimization strategy such as GA that will be discussed in next section.

### Optimization of control parameters

Strategies for solving old optimizing algorithm problems mostly depend on kind of aim, limit factors (linear, non-linear) and types of applied variation in sampling (true and natural). This is a fact that old optimizing approaches, causes limitations in solving mathematical programming and applied research approaches and this is mainly because of intrinsic solving mechanism in those approaches. One of the main features of old optimizing algorithms is their inflexibility for the supposed problem and it’s adaptation to possible and dynamic changes. Optimization means the way that tries to find the best parameter values in a function and in this paper the minimal deviation between actual and desired path must be found. For this purpose the classic strategies of optimal control can be used and by getting transfer function, the optimal parameters ((*M*_*d*_٫*K*_*d*_٫*D*_*d*_)٫*F*) are found to minimize the following cost function:

(14) $$\mathrm{Cost}\_F=\int_{0}^{n}e{\left(t\right)}^{2}\mathrm{dt}$$

Where *e*(*t*) is the deviation between actual and desired path and *n* is the number of stages in rehabilitation mode. Nevertheless, using these classic strategies result in more complexity of optimization problem and probably not finding a closed form answer owing to two reasons: firstly the feedback loop in block diagram is not identical for different cases (robotics kinematic are different). Secondly, because the parameters are in matrix form, an increase in their number, results in the increase of matrix dimensions. Therefore, defining an alternative strategy without the transfer function in order to minimize the cost function can be useful in decreasing complexity.

Eq. (15) is used for calculation of deviation between actual and desired path [20].

(15) ΔP=CF

Where *C* is the compliance matrix and it is defined as:

(16) $$C=J^{*}K^{-1}J^{*}{}^{T}$$

Where *K* is the stiffness matrix and J^*^ is defined as:

(17) $$\;J^{*}={\left(F^{-1}\right)}^{T}\tau^{T}$$

Now the impedance control parameters are modified so that cost function (18) can be minimized:

(18) Cost_F=‖ΔP‖

In this case, because of the interaction between robot and human, the amplitude of force *F* is very important and its high value can damage the patient. Therefore, the cost function is rewritten as:

(19) $$\mathrm{Cost}\_F=\min\left(\|,\Delta P\|\right)\;\mathrm{subject}\;\mathrm{to}\;\left(F\;\leq \;\mathrm{THRESHOLD}\left(\mathrm{Ft}\right)\right)$$

The threshold of force is changed based on the therapy of different stages and patient qualification. We can incorporate constraint of *F* in the cost function (19) and define a new cost function as:

(20) $$\mathrm{Cost}\_F=\mathrm{\alpha F}+\beta \left|\Delta P\right|,\;\left(\;\alpha +\beta =h,h\geq 1\right)\;$$

Where α٫β٫*h* are changed based on the therapy of different stages and patient improvement (adaptive strategy). *h* can be called the accuracy factor as larger values of *h* will result in higher accuracy. Now the control parameters such as (*M*_*d*_٫*K*_*d*_٫*D*_*d*_) and even *F* used for determination of necessary torques of links based on Eq. (3) are optimized by using a genetic evolutionary algorithm that will be explained in the next section.

### Genetic algorithm

GA is a multi-purpose search and optimization algorithm that is inspired by the theory of genetics and natural selection. The problem to be solved using GA is encoded as a chromosome that consists of several genes. The solution of the problem is represented by a group of chromosomes referred to as a population. In each iteration of the algorithm, the chromosomes in the population will undergo one or more genetic operations such as crossover and mutation. The result of the genetic operations will become the next generations of the solution. This process continues until either the solution is found or a certain termination condition is met. The idea behind GA is to have the chromosomes in the population to slowly converge to an optimal solution. At the same time, the algorithm is supposed to maintain enough diversity so that it can scan a large search space. It is the combination of these two characteristics that makes GA a good search and optimization algorithm.

In the suggested algorithm value representation is used and the cost function is considered as Eq. (20). The main goal is to reach the minimum level of (Δ*P*) considering (*F*) which will not be higher than the defined threshold. On the other hand, since the parameters are multi-dimensional, chromosomes will be multi-dimensional instead of being a linear vector. In this case, *M*_*d*_٫*K*_*d*_٫*D*_*d*_ and *F* will be the genes of each chromosome.

Thus, the chromosome length will be increased which in turn would result in the increase of problem complexity. For this reason, it is essential to find some techniques to decrease the chromosome length. Some of applicable techniques are:

•Converting the population of chromosomes to multi population.

•Fixing some of the parameters in any chromosome that are not very important or critical.

•Assuming the parameters of any chromosome as diagonal matrix.

In the first technique, the optimization of the whole parameters will not be done simultaneously and probably it will not result in the optimum result. The second technique is incoherence with the desired aim (adapting the controller parameters under the stability condition for different stroked patients and for different states of progression in the therapy process). Therefore, the third technique is applied in this study. And our mechanism for parent selection is truncation selection with this defined threshold in any generation.

T = average of Fitness_Function

Crossover operator is defined as one point crossover and point of crossover is selected randomly.

**Figure 3:**
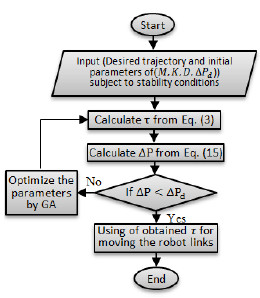
Flowchart of the suggested algorithm.

The flowchart of suggested algorithm is shown in Figure 3.

## Results and discussion

For implementation of the suggested algorithm on the planar 2-DOF robot described in previous sections, there are several requirements (in terms of position, joint torques, impedance parameters) needed to control the manipulator (MP) as the sequel:

1- Desired position and velocity (trajectory) of MP and Δ*P*_*d*_ obtained from the physiotherapist.

2- Finding the appropriate joint variables with desired trajectory based on IK implemented by NN.

3- Optimization of impedance control parameters using GA in order to determine the required torques.

All these requirements were discussed in the previous sections.

**Figure 4:**
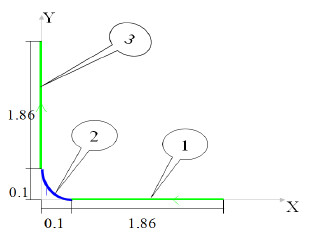
Trajectory in three phases.

For this purpose, a physiotherapy simple mode and its trajectory are defined which are shown in Figure 4.

The angles and velocities of joints for this robot are planned in three phases:

1) Horizontal trajectory from (***x٫*0**) to (***x***_***r***_٫**0)** with the speed of 1 m/s where *x* is the leg length in maximum extension and *x*_*r*_ is the distance between hip source and manipulator in minimum flexion in *x* direction (it is marked as the 1st phase in Figure 4).

2) Circular trajectory around hip from (***x***_***r***_٫**0)** to (**0٫*y***_***r***_) with the speed of 1 rad/s where ***y***_***r***_ is the distance between source and manipulator in minimum flexion in ***y*** direction (it is marked as 2nd phase in Figure 4).

3) Vertical trajectory from (**0٫*y***_***r***_) to (**0٫*y***) with speed of 1 m/s where ***y*** is the leg length in maximum extension (in it is marked as 3rd phase in Figure 4).

Assuming:

$$x=1.86,y=1.86,x_{r}=y_{r}=0.1$$

The velocities in three phases will be:

$$v_{1}=\left(-1,0\right)$$

$$\;v_{2}=\left(-\cos\left(10.t-18.6\right),\sin\left(10.t-18.6\right)\right)$$

$$v_{3}=\left(0,1\right)$$

Angles of the joints in this physiotherapy mode are obtained based on the equations of the inverse kinematic (IK) problem in the suggested 2-DOF planar robot:

(21) $$q_{2}=\mathrm{acos}\left(\frac{x^{2}+y^{2}-l_{1}^{2}-l_{2}^{2}}{2l_{1}l_{2}}\right)\;$$

(22) $$q_{1}=\mathrm{atan}2\left(\frac{y}{x}\right)-\mathrm{atan}2\left(\frac{l_{2}\sin\left(q_{2}\right)}{l_{1}+l_{2}\cos\left(q_{2}\right)}\right)$$

Taking into account the following:

(23) $$\left|\left(\frac{x^{2}+y^{2}-l_{1}^{2}-l_{2}^{2}}{2l_{1}l_{2}}\right)\right|\leq 1$$

**Figure 5:**
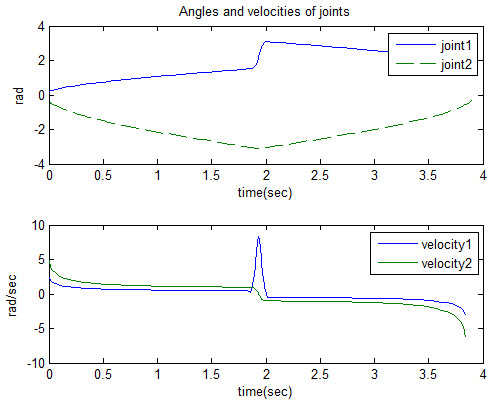
Angles and velocities of joints.

And they are shown in Figure 5. This figure depicts three phases in desired trajectory described in Figure 4.

Where the range of joint2 is complementary of *q*_2_ in Eq. (2).

Now, the proposed MLP neural network is used for solving the IK problem. The weight and bias of the MLP neural network for joint1 approximation are obtained after training for 100 epochs which are shown in Table 1. In this table w{1,1} and bi{1} are the weights and biases of layer (1), respectively and w{2,1} and bi{2} are the weights and bias of layer (2), respectively.

**Table 1:** Weight and bias of proposed MLP neural network for joint1 approximation

**w(1,1)**	**w(2,1)**	**bi(1)**	**bi(2)**
−1.3370	−2.7945	2.3883	0.4767
0.1686	−0.6542	−0.3739	
0.1437	−0.3735	−0.1026	
0.1332	−0.2878	−0.0565	
0.1914	−0.8640	−0.6795	
0.9674	2.5398	0.0812	
1.1624	−0.6009	−1.2252	
−0.1694	0.6654	0.3890	
−0.1473	0.4066	0.1242	
0.1583	−0.5220	−0.2194	
3.0389	−1.9478	−5.9277	
−0.0881	1.4560	−1.0628	
−0.1891	0.8422	0.6456	
0.1916	−0.8684	−0.6839	
0.1392	−0.3344	−0.0800	
0.1100	−0.1665	−0.0099	
−0.1663	0.6228	0.3329	
−1.0580	0.0915	1.6298	
11.9988	1.5388	−23.2530	

**Figure 6:**
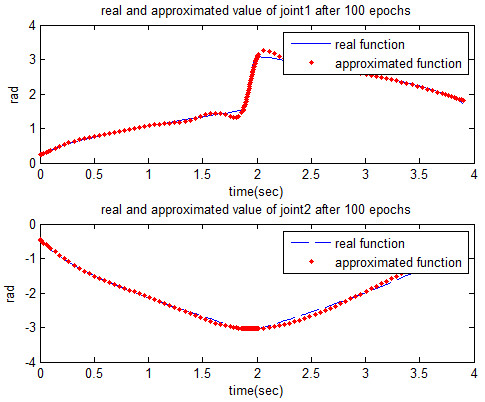
Approximated joint angles that produced from MLP network after 100 epochs.

**Figure 7:**
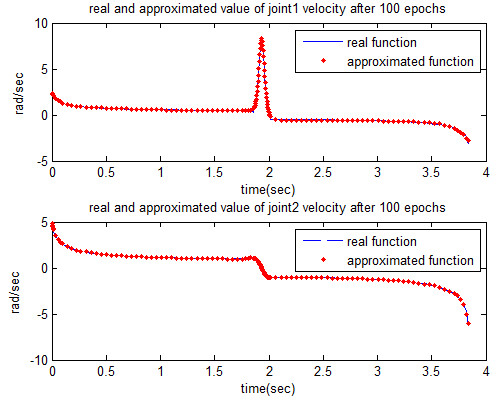
Approximated joint velocities that obtained from MLP network after 100 epochs.

The estimated values are shown in Figures 6 and 7, respectively.

The impedance parameters *K*_*d*_*, D*_*d*_*,M*_*d*_ are initially selected by a trial and error method subject to stability conditions and then they are tuned using GA algorithm. These parameters are chosen as below:

$$K_{d}=\mathrm{diag}\left(\mathrm{Ks}\right),D_{d}=\mathrm{diag}\left(\mathrm{Ds}\right),M_{d}=\mathrm{diag}\left(\mathrm{Mds}\right)$$

where the initial parameters are:

$$\mathrm{Ks}=0.05\;\left(\frac{N}{m}\right),\mathrm{Ms}=0.05\;\mathrm{kg},\mathrm{Ds}=0.05\left(\frac{\mathrm{Ns}}{m}\right)$$

If we consider Δ*P*_*d*_ = 10 *cm* and the characteristics of robot links as:

$$l\left(\mathrm{robot}\;\mathrm{arms}\right)=1m,$$

$$\;m_{1}=0.7\;\mathrm{kg},\;m_{2}=0.5\;\mathrm{kg}\;\left(\mathrm{link}\;\mathrm{weight}\right)$$

T=0.01sec⋅⋅⋅⋅R=0.001,C=0.1,b=400

**Figure 8:**
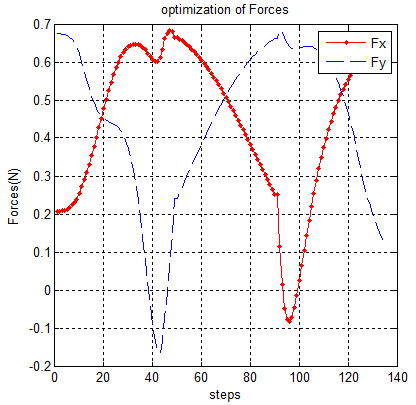
Forces on manipulator.

**Figure 9:**
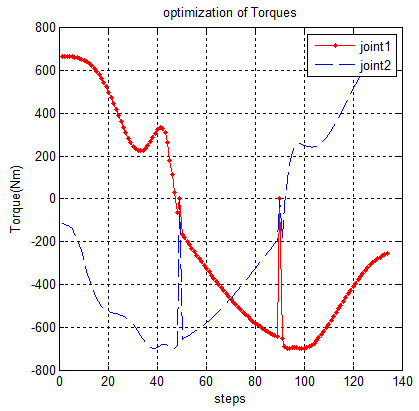
Torques on the joints.

The forces and torques that will be used for moving manipulator on the desired path (for 140 points of trajectory in 4 sec) are shown in Figures 8 and 9, respectively.

The evident peaks in Figure 9 denote the complementary movement.

The final optimized control parameters based on related torques and forces obtained as follows:

$$\begin{array}{l} \mathrm{Ms}=51.2\;\left(\frac{N}{m}\right),\mathrm{Mds}=4.8828*10^{-5}\;\mathrm{kg},\mathrm{Ds}\\ \;=0.05\left(\frac{\mathrm{Ns}}{m}\right) \end{array}$$

**Figure 10:**
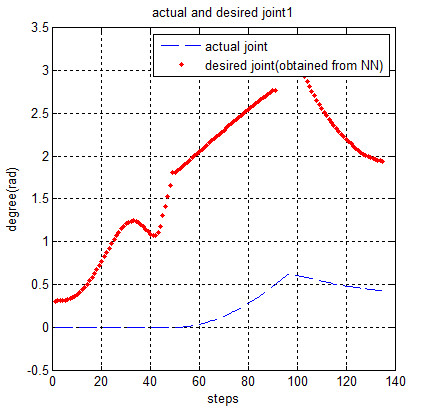
The actual and desired joint2.

**Figure 11:**
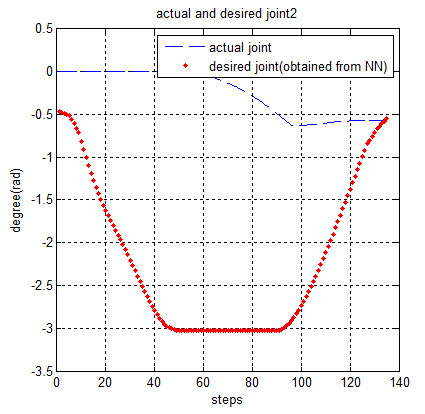
The actual and desired joint1.

Now, the obtained torques from Figure 9 are used for moving the robot. Figures 10 and 11 show the actual (*q*) and desired joint angles. It should be noted that the desired joint angles were approximated by MLP neural network that have been already described.

According to Figures 10 and 11 the deviation or difference between desired and actual variables is large for the first stages which are not suitable for rehabilitation without supervision. The deviation becomes smaller and converges to zero with the progress of simulation steps.

**Figure 12:**
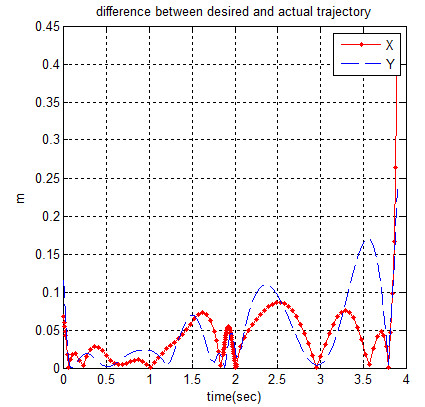
Difference between desired and actual trajectory.

The difference between the desired and actual trajectory is shown in Figure 12.

**Figure 13:**
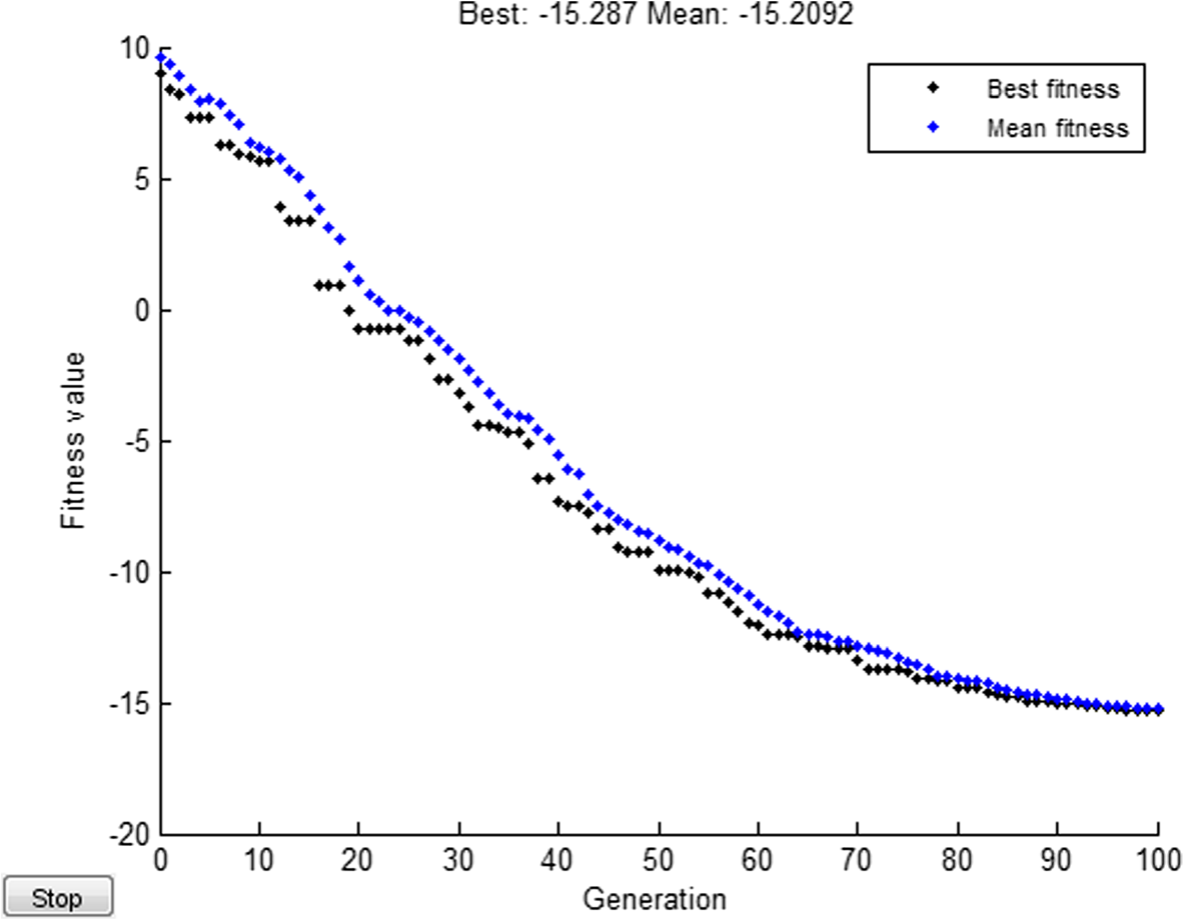
Fitness function diagram of GA.

Finally, fitness function diagram of GA for Eq. (20) is displayed in Figure 13.

And the optimal values for GA parameters are obtained as:

α=1.466⋅⋅⋅⋅,⋅⋅⋅⋅β⋅=⋅2.006

As the Figure 13 shows, the best control parameters obtained after 100 generations and the fitness function value is: -15.287.

## Conclusions

In this study, a therapeutic exercise planar 2-DOF robot was designed and controlled for lower-limb rehabilitation. The robot manipulator was controlled by combination of hybrid and adaptive controls. Some safety factors and stability constraints were defined and obtained. The robot is stopped when the safety factors are not satisfied. Kinematics of robot is estimated by an MLP neural network and proper control parameters are achieved using GA optimization.

The advantages of the proposed algorithm can be classified as the following:

1. The system is capable of learning the action of the physiotherapist for each patient and imitating this behavior in the absence of a physiotherapist that can be called robotherapy.

2. Generation of the source path is completely deliberative and it is done in accordance with the patient’s condition and the therapy’s duration. In this research, the source path was specified after various efforts such as visiting the specialists of the physiotherapy and observing several sessions in that section to completely gather the whole required information.

3. The neural network identifiers were used for solving the inverse kinematic of robot. The first idea for using NN is to cope with a non-linear identification problem and the second, more important one, is that the patient’s joints controlling system can be probably replaced by the artificial neural network.

4. Safety is guaranteed since some of the controller parameters can be adapted under the stability condition for different stroked patients and for different states of progression in the therapy process.

5. To reduce the complexity of optimization of control parameters, genetic evolution method was used. A different aspect of the defined chromosomes in the suggested algorithm in comparison to conventional methods is that they are defined as matrices not as vectors which were placed because of the abundance of DOF for a system.

In comparison to other related works, some other remarkable issues can be added as follows:

6. The work places that are needed for LOKOMAT [5] and LOPES [7] must be in a large room while the whole place that is needed for manufactured robot is 1 m^2^ in maximum. Moreover, the cost of rehabilitation with LOKOMAT is very high.

7. The number of DOF in ALEX [6] is very high but in 2-DOF planar robot it is limited to 2.

8. Only two parameters regarding the patient are used for starting the rehabilitation including mass of patient and ability in posture of ankle on the MP. The other parameters such as patient muscles, length and posture of whole body are not required.

## Abbreviations

CPM: Continues passive motions; GA: Genetic algorithm; MLP: Multi layer perceptron; MP: Manipulator; IK: Inverse kinematic.

## Competing interests

The authors declare that they have no competing interests.

## Authors’ contributions

FN proposed the design. WAA implemented the design on a manufactured 2–DOF robot. MAN worked on the optimization. All authors read and approved the final manuscript.

## Authors’ information

Wahab Amini Azar, received his B.Sc. in 1999 in Computer Engineering from the Shahid Beheshti University, Tehran, Iran and his M.Sc. in 2002 in Computer Engineering from the Amir Kabir University, Tehran, Iran. And currently he is Ph.D. student at Islamic Azad University, sciences and research branch,Tehran, Iran. Farid Najafi, received his B.Sc. in Mechanical Engineering From Department of Mechanical Engineering, Sharif University of Technology, Iran, and his M.Sc. and Ph.D. degrees From Faculty of Robotics and Automation, Moscow State Technical University (Bauman), Russia (in 2006). Now, he is associate professor of mechanical engineering at Guilan University, Rasht, Iran and working in the field of automatic control, robotics and mechatronic systems. Mohammad Ali Nekoui received his M.Sc. degree in Electrical Engineering from the University of Tehran in 1976, Diplome d’Espe cialisation in Instrume-ntation etMetrologie from Ecole Superieur d’Electricite (SUPEL EC), France, in 1979 and his Ph.D. degree at the School of Electrical and Electronic Engineering in Computer and Control Department from University of Leeds, U.K. in 1997. Since 1980, he has been with the K.N.Toosi. University of Technology. At present he is an Assistant Professor at the Faculty of Electrical and Computer Engineering of this university. His interests include linear and nonlinear optimization, linear systems, optimal control, and different aspects of mathematics in control.
